# Editorial: The RNA Revolution in Embryonic Development and Cell Differentiation in Health and Disease

**DOI:** 10.3389/fcell.2021.715341

**Published:** 2021-09-14

**Authors:** Alessandro Rosa, Constance Ciaudo, Pavel Sumazin, Francesco Fazi

**Affiliations:** ^1^Center for Life Nano Science, Istituto Italiano di Tecnologia, Rome, Italy; ^2^Laboratory Affiliated With Istituto Pasteur Italia-Fondazione Cenci Bolognetti, Department of Biology and Biotechnologies Charles Darwin, Sapienza University of Rome, Rome, Italy; ^3^Department of Biology, Swiss Federal Institute of Technology Zurich, Institute of Molecular Health Sciences (IMHS), Zurich, Switzerland; ^4^Texas Children's Cancer Center, Baylor College of Medicine, Houston, TX, United States; ^5^Department of Anatomical, Histological, Forensic and Orthopedic Sciences, Section of Histology and Medical Embryology, Sapienza University of Rome, Laboratory Affiliated With Istituto Pasteur Italia-Fondazione Cenci Bolognetti, Rome, Italy

**Keywords:** non-coding RNA (ncRNA), differentiation, microRNA (miRNA), circRNA, argonaute (AGO), embryonic development, RNA binding protein, long non-coding RNA (lncRNA)

## Introduction

Non-coding RNAs (ncRNAs) and their RNA binding proteins (RBPs) are emerging as crucial molecular players involved in normal and pathological cell fate determination. Among them microRNA (miRNAs), acting as gene expression regulators at post-transcriptional level, are involved in several networks relevant for the regulation of stemness, pluripotency, and cell fate determination during embryogenesis and adult life. Interestingly, the relevance for comprehension of the generation of spatio-temporal specificity of miRNA, their levels and dynamics of expression, and how the animal miRNA repertoire has evolved and diversified, is reported by Dexheimer and Cochella. The understanding of these regulation mechanisms could really help us to understand the contribution of miRNAs to the embryonic development and cell differentiation. Of note, alteration of the molecular mechanisms involving the ncRNA and their effectors in addition to defects in RNA modification and editing may contribute to the pathological cell fate determination in cancer and degenerative diseases.

## ncRNAs and RBPs in Development

How cells are able to maintain their identity or change fate specification is a complex problem in biology. The first events of cell fate specification take place during early development and are controlled at multiple levels by non-coding RNAs and RBPs. In the review from Müller et al., the authors discussed the importance of the Argonaute proteins for mammalian development and the recent discovery and implication of their post-translational modifications in the cancer context. Interestingly, these RBPs have functional relevance for miRNA gene regulation but also through their protein-protein interaction mediated *via* specific post-translational modifications. Another example of an RBP, IGF2BP3, is highlighted in an oncogenic context by Mancarella and Scotlandi, demonstrating that many RBPs are involved in several steps of the RNA life cycle. Indeed, in order to assess transcriptome-wide synthesis, processing, and degradation rates of RNAs, novel methodologies are now available, and Biasini and Marques present in this issue a detailed protocol to achieve metabolic labeling of RNA coupled with sequencing in mouse embryonic stem cells. Stem cells represent an attractive model to mimic early development *in vitro* and such cellular models are very important to allow reproducible research. Many cell types are still nowadays difficult to culture *ex vivo* and a better understanding at the molecular level of cues essential for the maintenance of their cellular identity is essential. Here, the work of Gao et al. unrevealed the role of the TGF-beta/Smad pathway in the production of adult beta cells by regulating the level of specific miRNAs, which is important for the medialization of type 1 diabetes.

## ncRNAs in the Pathophysiology of Muscle

ncRNAs are involved in the fine-tuning of gene expression at the basis of tissues homeostasis and regeneration. As reported by Martone et al. specific long ncRNAs (lncRNAs) and circRNAs are able to sustain skeletal and cardiac muscle formation. Of note, together with these ncRNAs, the contribution of Piwi-interacting RNAs (pi)RNAs and tRNA-derived fragments (tRFs) to myogenesis is also emerging. Interestingly, in this review it was discussed not only the nuclear and cytoplasmic activity of selected lncRNAs but also their ability to generate bioactive micropeptides which have been involved in specific muscle-related functions.

As reported by Sweta et al., recent studies suggest that lncRNAs are important for mesodermal specification and further differentiation, development, and function of mesodermal derivatives as skeletal muscle and cardiac lineages. The comprehension of the contribution of lncRNAs to cardiac, endothelial, and vascular smooth muscle cell function could be relevant to highlight how these molecules contribute to cardiac diseases and could be exploited as potential biomarkers. Regarding the clinical impact of ncRNAs for the muscle-related diseases as reported by Donzelli et al. miRNAs are emerging as potential cancer biomarkers in tissues and in body fluids for the early diagnosis of the cancer-related cachexia, a complex metabolic syndrome characterized by a marked reduction in muscle mass.

## ncRNAs in Neurological Disease

Regulatory ncRNAs, including miRNAs, lncRNAs and circular RNAs (circRNAs), are particularly enriched in the nervous system, where they play important roles in development, plasticity, and function. Moreover, increasing evidence links ncRNAs to aging, neurological diseases and brain tumors. Salvatori et al. focus on neural lncRNAs and circRNAs, reviewing the literature on their role in regulating neuronal and glial cells differentiation and their involvement in neurodegenerative diseases. The authors highlight the usefulness of ncRNAs as circulating biomarkers in body fluids and the possibility to use them as therapeutic molecules. Development of therapeutic approaches based on antisense oligonucleotides targeting ncRNAs is also discussed. The review by Laneve and Caffarelli concerns the contribution of ncRNAs to medulloblastoma, a common and aggressive pediatric brain tumor. In particular, the authors present extensive evidence on the roles played by miRNAs and lncRNAs as oncogenes or tumor suppressors, together with novel findings on other ncRNA classes, including circRNAs, enhancer RNAs (eRNAs), and small nuclear RNA. Deep characterization of such vast array of RNA species is particularly relevant in the context of medulloblastoma, as it can help decoding the intrinsically heterogeneous character of this severe brain tumor.

## ncRNAs in Cancer and Anti-Tumor Response

Long and short ncRNA dysregulation has been implicated with all stages of tumor genesis, progression, and therapeutic responses, with some RNAs acting in tumor-specific manners, and others dysregulated and affecting tumorigenesis in a pan-cancer fashion. Turco et al. reviewed the cancer genes and pathways that are affected by upregulation of members of the miRNA family miR-15/107 in multiple tumor types and discussed the diagnostic opportunities presented by the detection of these miRNAs in the blood of breast cancer patients. Tran et al. reviewed the dysregulation of circRNAs in gynecological and breast cancers and discussed the potential effects of circRNA dysregulation. Di Agostino et al. reviewed the regulatory roles of circRNAs in cancer and normal cells and argued that their dysregulation during cell maturation can lead to tumorigenesis, and, importantly, the formation of cancer stem cells, which may be responsible for therapeutic failures in multiple tumor types. The review by Mancarella and Scotlandi compiled evidence that suggests that the dysregulation of RBPs can contribute to aberrant ncRNA biogenesis in cancer, while the review by Fazi and Fatica collected evidence that aberrant N6-Methyladenosine (m^6^A) modifications can affect both the biogenesis and function of short and long RNAs in multiple tumor types. Finally, Pesce et al. reviewed the evidence for miRNA regulation of Natural Killer (NK) cell functions and their potential use as predictive biomarkers and effectors of immunotherapies. Their conclusions pointed to new opportunities for miRNA inhibitors and mimics as components in combination therapy strategies for cancer.

## Author Contributions

AR, CC, PS, and FF equally contributed as Guest Editors of this Research Topic and closely interacted throughout the editorial process, by defining the subjects to be treated and inviting leaders in specific research fields to contribute their work, and by acting as handling editors of the manuscripts submitted to the Research Topic and writing the Editorial. All authors contributed to the article and approved the submitted version.

## Funding

AR and FF were supported from research project grants from Sapienza University of Rome. PS was supported by the European Union's Horizon 2020 research and innovation programme under grant agreement 826121 and NCI award R21CA223140. CC was supported by a Swiss National Science Foundation grant (31003A_173120).

## Conflict of Interest

The authors declare that the research was conducted in the absence of any commercial or financial relationships that could be construed as a potential conflict of interest.

## Publisher's Note

All claims expressed in this article are solely those of the authors and do not necessarily represent those of their affiliated organizations, or those of the publisher, the editors and the reviewers. Any product that may be evaluated in this article, or claim that may be made by its manufacturer, is not guaranteed or endorsed by the publisher.

